# 

**Published:** 1869-04

**Authors:** 


### WESTERN NEW YORK DENTAL ASSOCIATION.

The Seventh semi-annual meeting of this Association will be held in the Court House, in the city of Rochester, commencing on Tuesday, May 4th, 1869, at ten o'clock in the forenoon, and continuing two days. It is expected that all members friendly to Dental education and improvement, will be present. There will be Clinics in Operative and Mechanical Dentistry. Demonstrations of new and improved methods of practice, and discussions of questions in Operative and Mechanical Dentistry, and in Dental Therapeutics. The Medical Profession are cordially invited to be present and join in the discussions.

REGULAR SUBJECTS FOR DISCUSSION.

*Nitrous Oxyd Gas and other Anæsthetics*—Their comparative merits. Essayist, A. P. Southwick.
*Improvements in Operative Dentistry*—Essayist, J. Requa.
*Mechanical Dentistry*—Essayist, M. B. Straight.
*Diseases of the Gums*—Essayist, B. T. Whitney.
*Miscellaneous Subjects*—Essayist, L. W. Bristol.

Frank French, *President.*

W. C. Barrett, *Secretary.*

*Warsaw*, March 15, 1869.

The regular meeting of the Seventh District Dental Society will be held at the same place on Tuesday, May 4th, 1869, commencing at 8 o'clock, P. M.

J. L. Clark, *Secretary.*

---

### NORTH IOWA DENTAL ASSOCIATION.

Dubuque, March 8th, 1869.

At the last annual meeting of the North Iowa Dental Association, it was *Resolved:* That the next annual meeting be held in Dubuque, on the second Tuesday in June, at 4 o'clock, P. M., and that the Executive Committee be instructed to issue a circular containing a list of subjects for discussion, so that members may come prepared to take part in the discussions. In conformity with the above resolution the committee have appointed the following list of subjects:

*Alveolar Abscess*—Dr. E. L. Clark, Dubuque.
*Filling Pulp Cavities*—Dr. A. B. Mason, Waterloo.
*Pathology of Dental Decay*—Dr. J. T. Abbott, Manchester.
*Salivary Calculus*—Dr. J. D. Russell, Vinton.
*Filling Teeth*—Dr. D. H. Gill, Independence.
*Mechanical Dentistry*—Dr. C. Poor, Dubuque.

J. S. Nicholson,

M. D. Goble,

*Executive Committee.*

### THE MAD RIVER DENTAL SOCIETY.

This Society meets at Oxford, Ohio, on Tuesday, May 18th. It is desirable that all the members be present; and a cordial invitation is extended to all other members of the profession who can make it *convenient to attend.*

### The Dental Register

### OF THE WEST.

*Issued Monthly, at $3 a Year in Advance.*

DEVOTED TO THE INTERESTS OF THE DENTAL PROFESSION.

EDITED BY J. TAFT AND G. WATT.

The volume commences in January and closes in December.

The Register being issued monthly is always fresh, therefore indispensable to the practitioner who desires to keep posted up with the new inventions and improvements which are daily developed. Neither expense nor effort will be spared to give value and variety to its contents. Articles will be illustrated with well executed engravings whenever they will add to the interest or assist in explanation.

Its extensive circulation and frequency of issue makes it one of the BEST DENTAL ADVERTISING MEDIUMS in the United States.

RATES OF ADVERTISING.

One page, 1 year, $50. Half page, 1 year, $30. Quarter page, or card, 1 year $20.

All advertising bills payable in advance, or at furthest after the issue of the first number containing the advertisement. All transient advertisements to be paid for in advance.

☞ CONTRIBUTIONS SOLICITED.

Address, J. TAFT, or "DENTAL REGISTER," Cincinnati Ohio.

Business Communications to

J. TAFT, Publisher,

No. 117 West Fourth St.

